# Supplementary material for: Regulation of Tomato Specialised Metabolism after Establishment of Symbiosis with the Endophytic Fungus Serendipita indica
Source: Microorganisms. 2022 Jan 16;10(1):194. doi: 10.3390/microorganisms10010194 (PMC8778627; doi:10.3390/microorganisms10010194)
Supplement: Supplementary file 1 [file microorganisms-10-00194-s001.zip › microorganisms-1533569-supplementary.pdf]

## Supplementary Tables

**Table S1.** Overview of the total number of clean reads generated after filtering and the read mapping rates to *S. lycopersicum* and *S. indica* genome. LS-Leaves from *S. indica*-inoculated plants, LC-Leaves from control plants, RS-Roots from *S. indica*-inoculated plants, RC-Roots from control plants

| Samples | Total clean reads | Mapped to *S. lycopersicum* genome[a] | Mapped to *S. indica* genome[b] | Mapping rate to *S. lycopersicum* gene models[c] |
|---|---|---|---|---|
| RC1 | 38227240 | 35487214 (92.83%) | 244 (0%) | 83.94% |
| RC2 | 47270758 | 43673880 (92.39%) | 8 (0%) | 83.96% |
| RC3 | 41130072 | 37796991 (91.9%) | 0 (0%) | 82.07% |
| RC4 | 46475282 | 42823753 (92.14%) | 0 (0%) | 83.16% |
| RS1 | 45532784 | 40862604 (89.74%) | 1195995 (2.63%) | 80.68% |
| RS2 | 48436740 | 42962249 (88.7%) | 1752964 (3.62%) | 79.55% |
| RS3 | 46513138 | 41191437 (88.56%) | 1575329 (3.39%) | 80.13% |
| RS4 | 45271188 | 40389016 (89.22%) | 1498744 (3.31%) | 80.61% |
| LC1 | 46198574 | 42966834 (93%) | 0 (0%) | 86.83% |
| LC2 | 53771394 | 49928827 (92.85%) | 0 (0%) | 86.52% |
| LC3 | 41846030 | 39049561 (93.32%) | 2 (0%) | 86.1% |
| LC4 | 44246664 | 41039108 (92.75%) | 0 (0%) | 86.15% |
| LS1 | 51285910 | 47739147 (93.08%) | 57168 (0.11%) | 86.06% |
| LS2 | 49844254 | 46353638 (93%) | 10252 (0.02%) | 85.84% |
| LS3 | 47361048 | 44102554 (93.12%) | 681 (0%) | 86.2% |
| LS4 | 48618546 | 45273435 (93.12%) | 2370 (0%) | 86.22% |

[a] Mapping to *S. lycopersicum* genome (release41, Ensembl) was performed using TopHat2 [86] from Novogene Bioinformatics Technology Co. Ltd (Hong Kong). [b] Mapping to *S. indica* genome (JGI genome portal) was performed using Bowtie 2 [87] from Novogene Bioinformatics Technology Co. Ltd (Hong Kong). [c] Mapping to *S. lycopersicum* gene models (ITAG3.2, SolGenomics) was performed with Salmon version 0.12.0 [40]

**Table S2.** GO terms enriched in statistically up-regulated genes (≥2-fold change) in leaves of tomato plants colonised by *S. indica*. Simulation corrected *P* ≤0.05.

| GO number | Gene Ontology term | Cluster frequency | Genome frequency of use | Corrected *P*-value |
|---|---|---|---|---|
| | unannotated | 30 out of 391 genes, 7.7% | 8 out of 34727 genes, 0.0% | <0.001 |
| GO:0042254 | ribosome biogenesis | 48 out of 391 genes, 12.3% | 1141 out of 34727 genes, 3.3% | <0.001 |
| GO:0006364 | rRNA processing | 41 out of 391 genes, 10.5% | 1027 out of 34727 genes, 3.0% | <0.001 |
| GO:0016072 | rRNA metabolic process | 41 out of 391 genes, 10.5% | 1048 out of 34727 genes, 3.0% | <0.001 |
| GO:0009220 | pyrimidine ribonucleotide biosynthetic process | 21 out of 391 genes, 5.4% | 272 out of 34727 genes, 0.8% | <0.001 |
| GO:0009218 | pyrimidine ribonucleotide metabolic process | 21 out of 391 genes, 5.4% | 283 out of 34727 genes, 0.8% | <0.001 |
| GO:0022613 | ribonucleoprotein complex biogenesis and assembly | 52 out of 391 genes, 13.3% | 1646 out of 34727 genes, 4.7% | <0.001 |
| GO:0006221 | pyrimidine nucleotide biosynthetic process | 21 out of 391 genes, 5.4% | 293 out of 34727 genes, 0.8% | <0.001 |
| GO:0030490 | maturation of SSU-rRNA | 14 out of 391 genes, 3.6% | 116 out of 34727 genes, 0.3% | <0.001 |
| GO:0006220 | pyrimidine nucleotide metabolic process | 21 out of 391 genes, 5.4% | 310 out of 34727 genes, 0.9% | <0.001 |
| GO:0034470 | ncRNA processing | 43 out of 391 genes, 11.0% | 1273 out of 34727 genes, 3.7% | <0.001 |
| GO:0000478 | endonucleolytic cleavages during rRNA processing | 10 out of 391 genes, 2.6% | 61 out of 34727 genes, 0.2% | <0.001 |
| GO:0000469 | cleavages during rRNA processing | 11 out of 391 genes, 2.8% | 85 out of 34727 genes, 0.2% | <0.001 |
| GO:0034660 | ncRNA metabolic process | 43 out of 391 genes, 11.0% | 1455 out of 34727 genes, 4.2% | <0.001 |
| GO:0000462 | maturation of SSU-rRNA from tricistronic rRNA transcript (SSU-rRNA, 5.8S rRNA, LSU-rRNA) | 10 out of 391 genes, 2.6% | 81 out of 34727 genes, 0.2% | <0.001 |
| GO:0000480 | endonucleolytic cleavage in 5'-ETS of tricistronic rRNA transcript (SSU-rRNA, 5.8S rRNA, LSU-rRNA) | 8 out of 391 genes, 2.0% | 45 out of 34727 genes, 0.1% | <0.001 |
| GO:0000472 | endonucleolytic cleavage to generate mature 5'-end of SSU-rRNA from (SSU-rRNA, 5.8S rRNA, LSU-rRNA) | 8 out of 391 genes, 2.0% | 49 out of 34727 genes, 0.1% | <0.001 |
| GO:0006606 | protein import into nucleus | 18 out of 391 genes, 4.6% | 342 out of 34727 genes, 1.0% | <0.001 |
| GO:0000967 | rRNA 5'-end processing | 8 out of 391 genes, 2.0% | 52 out of 34727 genes, 0.1% | <0.001 |
| GO:0034471 | ncRNA 5'-end processing | 8 out of 391 genes, 2.0% | 52 out of 34727 genes, 0.1% | <0.001 |
| GO:0000966 | RNA 5'-end processing | 8 out of 391 genes, 2.0% | 56 out of 34727 genes, 0.2% | <0.001 |
| GO:0051170 | nuclear import | 18 out of 391 genes, 4.6% | 367 out of 34727 genes, 1.1% | <0.001 |
| GO:0000479 | endonucleolytic cleavage of tricistronic rRNA transcript (SSU-rRNA, 5.8S rRNA, LSU-rRNA) | 8 out of 391 genes, 2.0% | 57 out of 34727 genes, 0.2% | <0.001 |
| GO:0006396 | RNA processing | 64 out of 391 genes, 16.4% | 2972 out of 34727 genes, 8.6% | <0.001 |
| GO:0009260 | ribonucleotide biosynthetic process | 21 out of 391 genes, 5.4% | 511 out of 34727 genes, 1.5% | <0.001 |

| | | | | |
|---|---|---|---|---|
| GO:0009451 | RNA modification | 28 out of 391 genes, 7.2% | 861 out of 34727 genes, 2.5% | 0.002 |
| GO:0051169 | nuclear transport | 24 out of 391 genes, 6.1% | 688 out of 34727 genes, 2.0% | 0.003 |
| GO:0006913 | nucleocytoplasmic transport | 23 out of 391 genes, 5.9% | 672 out of 34727 genes, 1.9% | 0.005 |
| GO:0009165 | nucleotide biosynthetic process | 29 out of 391 genes, 7.4% | 1062 out of 34727 genes, 3.1% | 0.019 |
| GO:0016554 | cytidine to uridine editing | 9 out of 391 genes, 2.3% | 142 out of 34727 genes, 0.4% | 0.049 |

**Table S3.** GO terms enriched in statistically down-regulated genes (>2-fold change) in leaves of tomato plants colonised by *S. indica*. Simulation corrected *P* ≤0.05.

| GO number | Gene Ontology term | Cluster frequency | Genome frequency of use | Corrected *P*-value |
|---|---|---|---|---|
| | unannotated | 36 out of 868 genes, 4.1% | 3 out of 34727 genes, 0.0% | <0.001 |
| GO:0003700 | transcription factor activity | 110 out of 868 genes, 12.7% | 2215 out of 34727 genes, 6.4% | <0.001 |
| GO:0016758 | transferase activity, transferring hexosyl groups | 49 out of 868 genes, 5.6% | 655 out of 34727 genes, 1.9% | <0.001 |
| GO:0035251 | UDP-glucosyltransferase activity | 32 out of 868 genes, 3.7% | 316 out of 34727 genes, 0.9% | <0.001 |
| GO:0046527 | glucosyltransferase activity | 32 out of 868 genes, 3.7% | 333 out of 34727 genes, 1.0% | <0.001 |
| GO:0047893 | flavonol 3-O-glucosyltransferase activity | 20 out of 868 genes, 2.3% | 136 out of 34727 genes, 0.4% | <0.001 |
| GO:0008194 | UDP-glycosyltransferase activity | 39 out of 868 genes, 4.5% | 490 out of 34727 genes, 1.4% | <0.001 |
| GO:0016757 | transferase activity, transferring glycosyl groups | 52 out of 868 genes, 6.0% | 830 out of 34727 genes, 2.4% | <0.001 |
| GO:0030528 | transcription regulator activity | 114 out of 868 genes, 13.1% | 2579 out of 34727 genes, 7.4% | <0.001 |
| GO:0050403 | trans-zeatin O-beta-D-glucosyltransferase activity | 16 out of 868 genes, 1.8% | 106 out of 34727 genes, 0.3% | <0.001 |
| GO:0050502 | cis-zeatin O-beta-D-glucosyltransferase activity | 11 out of 868 genes, 1.3% | 56 out of 34727 genes, 0.2% | <0.001 |
| GO:0008509 | anion transmembrane transporter activity | 28 out of 868 genes, 3.2% | 378 out of 34727 genes, 1.1% | <0.001 |
| GO:0015293 | symporter activity | 26 out of 868 genes, 3.0% | 341 out of 34727 genes, 1.0% | <0.001 |
| GO:0042937 | tripeptide transporter activity | 12 out of 868 genes, 1.4% | 82 out of 34727 genes, 0.2% | <0.001 |
| GO:0032795 | heterotrimeric G-protein binding | 5 out of 868 genes, 0.6% | 9 out of 34727 genes, 0.0% | <0.001 |
| GO:0042936 | dipeptide transporter activity | 12 out of 868 genes, 1.4% | 84 out of 34727 genes, 0.2% | <0.001 |
| GO:0015103 | inorganic anion transmembrane transporter activity | 20 out of 868 genes, 2.3% | 233 out of 34727 genes, 0.7% | <0.001 |
| GO:0016762 | xyloglucan:xyloglucosyl transferase activity | 8 out of 868 genes, 0.9% | 38 out of 34727 genes, 0.1% | <0.001 |
| GO:0015334 | high affinity oligopeptide transporter activity | 10 out of 868 genes, 1.2% | 65 out of 34727 genes, 0.2% | 0.001 |
| GO:0015291 | secondary active transmembrane transporter activity | 34 out of 868 genes, 3.9% | 594 out of 34727 genes, 1.7% | 0.002 |
| GO:0015198 | oligopeptide transporter activity | 12 out of 868 genes, 1.4% | 103 out of 34727 genes, 0.3% | 0.002 |
| GO:0015197 | peptide transporter activity | 12 out of 868 genes, 1.4% | 106 out of 34727 genes, 0.3% | 0.008 |
| GO:0004604 | phosphoadenylyl-sulfate reductase (thioredoxin) activity | 3 out of 868 genes, 0.3% | 3 out of 34727 genes, 0.0% | 0.013 |
| GO:0009973 | adenylyl-sulfate reductase activity | 3 out of 868 genes, 0.3% | 3 out of 34727 genes, 0.0% | 0.013 |
| GO:0033741 | adenylyl-sulfate reductase (glutathione) activity | 3 out of 868 genes, 0.3% | 3 out of 34727 genes, 0.0% | 0.013 |

| | | | | |
|---|---|---|---|---|
| GO:0031683 | G-protein beta/gamma-subunit binding | 5 out of 868 genes, 0.6% | 15 out of 34727 genes, 0.0% | 0.015 |
| GO:0016759 | cellulose synthase activity | 8 out of 868 genes, 0.9% | 56 out of 34727 genes, 0.2% | 0.046 |
| GO:0016760 | cellulose synthase (UDP-forming) activity | 8 out of 868 genes, 0.9% | 56 out of 34727 genes, 0.2% | 0.046 |
| GO:0022892 | substrate-specific transporter activity | 73 out of 868 genes, 8.4% | 1842 out of 34727 genes, 5.3% | 0.047 |

**Table S4.** GO terms enriched in statistically up-regulated genes (≥2-fold change) in tomato roots colonised by *S. indica*. Simulation corrected *P* ≤0.05.

| GO number | Gene Ontology term | Cluster frequency | Genome frequency of use | Corrected *P*-value |
|---|---|---|---|---|
| | unannotated | 7 out of 128 genes, 5.5% | 8 out of 34727 genes, 0.0% | <0.001 |
| GO:0006952 | defence response | 60 out of 128 genes, 46.9% | 6089 out of 34727 genes, 17.5% | <0.001 |
| GO:0006950 | response to stress | 84 out of 128 genes, 65.6% | 12568 out of 34727 genes, 36.2% | <0.001 |
| GO:0042742 | defence response to bacterium | 33 out of 128 genes, 25.8% | 2350 out of 34727 genes, 6.8% | <0.001 |
| GO:0009617 | response to bacterium | 38 out of 128 genes, 29.7% | 3151 out of 34727 genes, 9.1% | <0.001 |
| GO:0009607 | response to biotic stimulus | 56 out of 128 genes, 43.8% | 6463 out of 34727 genes, 18.6% | <0.001 |
| GO:0045087 | innate immune response | 43 out of 128 genes, 33.6% | 4265 out of 34727 genes, 12.3% | <0.001 |
| GO:0050896 | response to stimulus | 92 out of 128 genes, 71.9% | 15482 out of 34727 genes, 44.6% | <0.001 |
| GO:0006955 | immune response | 43 out of 128 genes, 33.6% | 4428 out of 34727 genes, 12.8% | <0.001 |
| GO:0009814 | defence response, incompatible interaction | 36 out of 128 genes, 28.1% | 3304 out of 34727 genes, 9.5% | <0.001 |
| GO:0051707 | response to other organism | 49 out of 128 genes, 38.3% | 5651 out of 34727 genes, 16.3% | <0.001 |
| GO:0002376 | immune system process | 44 out of 128 genes, 34.4% | 4830 out of 34727 genes, 13.9% | <0.001 |
| GO:0045088 | regulation of innate immune response | 30 out of 128 genes, 23.4% | 2611 out of 34727 genes, 7.5% | <0.001 |
| GO:0010200 | response to chitin | 28 out of 128 genes, 21.9% | 2359 out of 34727 genes, 6.8% | <0.001 |
| GO:0050776 | regulation of immune response | 30 out of 128 genes, 23.4% | 2749 out of 34727 genes, 7.9% | <0.001 |
| GO:0010363 | regulation of plant-type hypersensitive response | 28 out of 128 genes, 21.9% | 2447 out of 34727 genes, 7.0% | <0.001 |
| GO:0031347 | regulation of defence response | 31 out of 128 genes, 24.2% | 2971 out of 34727 genes, 8.6% | <0.001 |
| GO:0002682 | regulation of immune system process | 30 out of 128 genes, 23.4% | 2882 out of 34727 genes, 8.3% | <0.001 |
| GO:0009863 | salicylic acid mediated signalling pathway | 25 out of 128 genes, 19.5% | 2215 out of 34727 genes, 6.4% | <0.001 |
| GO:0006612 | protein targeting to membrane | 27 out of 128 genes, 21.1% | 2645 out of 34727 genes, 7.6% | 0.001 |
| GO:0051704 | multi-organism process | 51 out of 128 genes, 39.8% | 7325 out of 34727 genes, 21.1% | 0.001 |
| GO:0031640 | killing of cells of another organism | 8 out of 128 genes, 6.2% | 210 out of 34727 genes, 0.6% | 0.001 |

| | | | | |
|---|---|---|---|---|
| GO:0010033 | response to organic substance | 38 out of 128 genes, 29.7% | 4667 out of 34727 genes, 13.4% | 0.001 |
| GO:0009743 | response to carbohydrate stimulus | 34 out of 128 genes, 26.6% | 3915 out of 34727 genes, 11.3% | 0.001 |
| GO:0007242 | intracellular signalling cascade | 47 out of 128 genes, 36.7% | 6527 out of 34727 genes, 18.8% | 0.001 |
| GO:0001906 | cell killing | 8 out of 128 genes, 6.2% | 216 out of 34727 genes, 0.6% | 0.002 |
| GO:0009719 | response to endogenous stimulus | 47 out of 128 genes, 36.7% | 6589 out of 34727 genes, 19.0% | 0.002 |
| GO:0009751 | response to salicylic acid stimulus | 27 out of 128 genes, 21.1% | 2749 out of 34727 genes, 7.9% | 0.002 |
| GO:0046483 | heterocycle metabolic process | 26 out of 128 genes, 20.3% | 2617 out of 34727 genes, 7.5% | 0.002 |
| GO:0042445 | hormone metabolic process | 21 out of 128 genes, 16.4% | 1810 out of 34727 genes, 5.2% | 0.002 |
| GO:0042435 | indole derivative biosynthetic process | 13 out of 128 genes, 10.2% | 741 out of 34727 genes, 2.1% | 0.003 |
| GO:0042430 | indole and derivative metabolic process | 14 out of 128 genes, 10.9% | 863 out of 34727 genes, 2.5% | 0.003 |
| GO:0042434 | indole derivative metabolic process | 14 out of 128 genes, 10.9% | 863 out of 34727 genes, 2.5% | 0.003 |
| GO:0006725 | cellular aromatic compound metabolic process | 34 out of 128 genes, 26.6% | 4125 out of 34727 genes, 11.9% | 0.004 |
| GO:0009611 | response to wounding | 28 out of 128 genes, 21.9% | 3029 out of 34727 genes, 8.7% | 0.004 |
| GO:0006995 | cellular response to nitrogen starvation | 10 out of 128 genes, 7.8% | 434 out of 34727 genes, 1.2% | 0.004 |
| GO:0009627 | systemic acquired resistance | 24 out of 128 genes, 18.8% | 2374 out of 34727 genes, 6.8% | 0.004 |
| GO:0048583 | regulation of response to stimulus | 32 out of 128 genes, 25.0% | 3810 out of 34727 genes, 11.0% | 0.004 |
| GO:0042436 | indole derivative catabolic process | 10 out of 128 genes, 7.8% | 448 out of 34727 genes, 1.3% | 0.004 |
| GO:0009605 | response to external stimulus | 44 out of 128 genes, 34.4% | 6280 out of 34727 genes, 18.1% | 0.007 |
| GO:0043562 | cellular response to nitrogen levels | 10 out of 128 genes, 7.8% | 456 out of 34727 genes, 1.3% | 0.008 |
| GO:0031348 | negative regulation of defence response | 19 out of 128 genes, 14.8% | 1647 out of 34727 genes, 4.7% | 0.011 |
| GO:0042446 | hormone biosynthetic process | 18 out of 128 genes, 14.1% | 1499 out of 34727 genes, 4.3% | 0.011 |
| GO:0009753 | response to jasmonic acid stimulus | 26 out of 128 genes, 20.3% | 2848 out of 34727 genes, 8.2% | 0.012 |
| GO:0043067 | regulation of programmed cell death | 29 out of 128 genes, 22.7% | 3393 out of 34727 genes, 9.8% | 0.012 |
| GO:0007165 | signal transduction | 49 out of 128 genes, 38.3% | 7570 out of 34727 genes, 21.8% | 0.016 |
| GO:0006569 | tryptophan catabolic process | 9 out of 128 genes, 7.0% | 418 out of 34727 genes, 1.2% | 0.023 |
| GO:0046218 | indolalkylamine catabolic process | 9 out of 128 genes, 7.0% | 418 out of 34727 genes, 1.2% | 0.023 |
| GO:0010817 | regulation of hormone levels | 24 out of 128 genes, 18.8% | 2649 out of 34727 genes, 7.6% | 0.032 |
| GO:0009867 | jasmonic acid mediated signalling pathway | 20 out of 128 genes, 15.6% | 1965 out of 34727 genes, 5.7% | 0.032 |
| GO:0009074 | aromatic amino acid family catabolic process | 9 out of 128 genes, 7.0% | 449 out of 34727 genes, 1.3% | 0.047 |
| GO:0002679 | respiratory burst during defence response | 11 out of 128 genes, 8.6% | 682 out of 34727 genes, 2.0% | 0.049 |

| | | | | |
|---|---|---|---|---|
| GO:0042221 | response to chemical stimulus | 62 out of 128 genes, 48.4% | 10951 out of 34727 genes, 31.5% | 0.05 |

**Table S5.** GO terms enriched in statistically down-regulated genes (≥2-fold change) in tomato roots colonised by *S. indica*. Simulation corrected *P* ≤0.05.

| GO number | Gene Ontology term | Cluster frequency | Genome frequency of use | Corrected *P*-value |
|---|---|---|---|---|
| GO:0009416 | response to light stimulus | 58 out of 172 genes, 33.7% | 4203 out of 34727 genes, 12.1% | <0.001 |
| | unannotated | 6 out of 172 genes, 3.5% | 8 out of 34727 genes, 0.0% | <0.001 |
| GO:0009314 | response to radiation | 58 out of 172 genes, 33.7% | 4446 out of 34727 genes, 12.8% | <0.001 |
| GO:0010218 | response to far red light | 18 out of 172 genes, 10.5% | 431 out of 34727 genes, 1.2% | <0.001 |
| GO:0010114 | response to red light | 18 out of 172 genes, 10.5% | 444 out of 34727 genes, 1.3% | <0.001 |
| GO:0009765 | photosynthesis, light harvesting | 9 out of 172 genes, 5.2% | 59 out of 34727 genes, 0.2% | <0.001 |
| GO:0009639 | response to red or far red light | 29 out of 172 genes, 16.9% | 1456 out of 34727 genes, 4.2% | <0.001 |
| GO:0009637 | response to blue light | 16 out of 172 genes, 9.3% | 397 out of 34727 genes, 1.1% | <0.001 |
| GO:0018298 | protein-chromophore linkage | 10 out of 172 genes, 5.8% | 146 out of 34727 genes, 0.4% | <0.001 |
| GO:0009628 | response to abiotic stimulus | 78 out of 172 genes, 45.3% | 8617 out of 34727 genes, 24.8% | <0.001 |
| GO:0009698 | phenylpropanoid metabolic process | 28 out of 172 genes, 16.3% | 1650 out of 34727 genes, 4.8% | <0.001 |
| GO:0019748 | secondary metabolic process | 46 out of 172 genes, 26.7% | 4093 out of 34727 genes, 11.8% | <0.001 |
| GO:0050896 | response to stimulus | 111 out of 172 genes, 64.5% | 15482 out of 34727 genes, 44.6% | <0.001 |
| GO:0042221 | response to chemical stimulus | 87 out of 172 genes, 50.6% | 10951 out of 34727 genes, 31.5% | <0.001 |
| GO:0009699 | phenylpropanoid biosynthetic process | 23 out of 172 genes, 13.4% | 1311 out of 34727 genes, 3.8% | <0.001 |
| GO:0042440 | pigment metabolic process | 23 out of 172 genes, 13.4% | 1371 out of 34727 genes, 3.9% | <0.001 |
| GO:0006575 | amino acid derivative metabolic process | 34 out of 172 genes, 19.8% | 2805 out of 34727 genes, 8.1% | 0.001 |
| GO:0009812 | flavonoid metabolic process | 19 out of 172 genes, 11.0% | 1085 out of 34727 genes, 3.1% | 0.002 |
| GO:0043647 | inositol phosphate metabolic process | 8 out of 172 genes, 4.7% | 173 out of 34727 genes, 0.5% | 0.003 |
| GO:0043455 | regulation of secondary metabolic process | 21 out of 172 genes, 12.2% | 1319 out of 34727 genes, 3.8% | 0.003 |
| GO:0009642 | response to light intensity | 18 out of 172 genes, 10.5% | 999 out of 34727 genes, 2.9% | 0.003 |
| GO:0015698 | inorganic anion transport | 22 out of 172 genes, 12.8% | 1448 out of 34727 genes, 4.2% | 0.003 |
| GO:0006725 | cellular aromatic compound metabolic process | 42 out of 172 genes, 24.4% | 4125 out of 34727 genes, 11.9% | 0.003 |
| GO:0009744 | response to sucrose stimulus | 18 out of 172 genes, 10.5% | 1048 out of 34727 genes, 3.0% | 0.003 |
| GO:0034285 | response to disaccharide stimulus | 18 out of 172 genes, 10.5% | 1048 out of 34727 genes, 3.0% | 0.003 |
| GO:0006629 | lipid metabolic process | 50 out of 172 genes, 29.1% | 5426 out of 34727 genes, 15.6% | 0.003 |

| | | | | |
|---|---|---|---|---|
| GO:0010264 | myo-inositol hexakisphosphate biosynthetic process | 7 out of 172 genes, 4.1% | 137 out of 34727 genes, 0.4% | 0.003 |
| GO:0033517 | myo-inositol hexakisphosphate metabolic process | 7 out of 172 genes, 4.1% | 137 out of 34727 genes, 0.4% | 0.003 |
| GO:0010033 | response to organic substance | 45 out of 172 genes, 26.2% | 4667 out of 34727 genes, 13.4% | 0.003 |
| GO:0019684 | photosynthesis, light reaction | 15 out of 172 genes, 8.7% | 765 out of 34727 genes, 2.2% | 0.004 |
| GO:0015979 | photosynthesis | 17 out of 172 genes, 9.9% | 970 out of 34727 genes, 2.8% | 0.004 |
| GO:0019751 | polyol metabolic process | 9 out of 172 genes, 5.2% | 265 out of 34727 genes, 0.8% | 0.004 |
| GO:0032958 | inositol phosphate biosynthetic process | 7 out of 172 genes, 4.1% | 146 out of 34727 genes, 0.4% | 0.004 |
| GO:0006020 | inositol metabolic process | 8 out of 172 genes, 4.7% | 205 out of 34727 genes, 0.6% | 0.004 |
| GO:0042754 | negative regulation of circadian rhythm | 3 out of 172 genes, 1.7% | 9 out of 34727 genes, 0.0% | 0.007 |
| GO:0043496 | regulation of protein homodimerization activity | 3 out of 172 genes, 1.7% | 9 out of 34727 genes, 0.0% | 0.007 |
| GO:0009963 | positive regulation of flavonoid biosynthetic process | 14 out of 172 genes, 8.1% | 692 out of 34727 genes, 2.0% | 0.007 |
| GO:0006021 | inositol biosynthetic process | 7 out of 172 genes, 4.1% | 149 out of 34727 genes, 0.4% | 0.007 |
| GO:0006631 | fatty acid metabolic process | 27 out of 172 genes, 15.7% | 2202 out of 34727 genes, 6.3% | 0.009 |
| GO:0009644 | response to high light intensity | 14 out of 172 genes, 8.1% | 713 out of 34727 genes, 2.1% | 0.013 |
| GO:0009813 | flavonoid biosynthetic process | 17 out of 172 genes, 9.9% | 1035 out of 34727 genes, 3.0% | 0.015 |
| GO:0046173 | polyol biosynthetic process | 7 out of 172 genes, 4.1% | 163 out of 34727 genes, 0.5% | 0.015 |
| GO:0010155 | regulation of proton transport | 8 out of 172 genes, 4.7% | 234 out of 34727 genes, 0.7% | 0.02 |
| GO:0042398 | amino acid derivative biosynthetic process | 26 out of 172 genes, 15.1% | 2161 out of 34727 genes, 6.2% | 0.02 |
| GO:0009725 | response to hormone stimulus | 51 out of 172 genes, 29.7% | 5884 out of 34727 genes, 16.9% | 0.024 |
| GO:0048572 | short-day photoperiodism | 6 out of 172 genes, 3.5% | 116 out of 34727 genes, 0.3% | 0.026 |
| GO:0006820 | anion transport | 22 out of 172 genes, 12.8% | 1677 out of 34727 genes, 4.8% | 0.028 |
| GO:0009719 | response to endogenous stimulus | 55 out of 172 genes, 32.0% | 6589 out of 34727 genes, 19.0% | 0.028 |
| GO:0044255 | cellular lipid metabolic process | 45 out of 172 genes, 26.2% | 4970 out of 34727 genes, 14.3% | 0.028 |
| GO:0048575 | short-day photoperiodism, flowering | 5 out of 172 genes, 2.9% | 74 out of 34727 genes, 0.2% | 0.034 |
| GO:0009648 | photoperiodism | 9 out of 172 genes, 5.2% | 329 out of 34727 genes, 0.9% | 0.041 |
| GO:0046148 | pigment biosynthetic process | 17 out of 172 genes, 9.9% | 1123 out of 34727 genes, 3.2% | 0.043 |
| GO:0006520 | cellular amino acid and derivative metabolic process | 39 out of 172 genes, 22.7% | 4118 out of 34727 genes, 11.9% | 0.043 |

**Table S6.** Key genes involved in the biosynthesis of sterols and glycoalkaloids in tomato plants. LS-Leaves from *S. indica*-inoculated plants, LC-Leaves from control plants, RS-Roots from *S. indica*-inoculated plants, RC-Roots from control plants, Padj represents the FDR corrected *P*-value [47].

| **geneID** | **Annotation** | **log2FoldChange (LSvsLC)** | **Padj (LSvsLC)** | **log2FoldChange (RSvsRC)** | **Padj (RSvsRC)** |
|---|---|---|---|---|---|
| Solyc03g032010.3.1 | HMGR1 | -1,071862292 | 2,71E-12 | 0,103935545 | 0,464283426 |
| Solyc01g110290.3.1 | SQS | -0,345804369 | 0,007375011 | -0,032854916 | 0,760343841 |
| Solyc12g006530.2.1 | TTS1 | -1,955851209 | 0,115840374 | -1,276454187 | 0,011412626 |
| Solyc12g006520.2.1 | TTS2 | 0,015512455 | 0,993705338 | 0,090562205 | 0,743155339 |
| Solyc04g070980.3.1 | CAS1 | -0,292381932 | 0,193009074 | -0,041647917 | 0,798240379 |
| Solyc01g087560.3.1 | SMT1 | 0,141969113 | 0,597019637 | 0,048563191 | 0,763076686 |
| Solyc02g069490.3.1 | SSR2 | -1,431619619 | 8,38E-16 | -0,237390468 | 0,146353048 |
| Solyc02g086180.3.1 | C5-SD | -1,93644664 | 2,22E-09 | -0,142034096 | 0,630482937 |
| Solyc07g043420.3.1 | GAME11 | -1,503566951 | 9,14E-09 | -0,151616616 | 0,52471162 |
| Solyc12g006460.2.1 | GAME4 | -0,815255056 | 1,04E-05 | -0,196100414 | 0,235506181 |
| Solyc12g006470.2.1 | GAME12 | -0,863429161 | 0,000111696 | -0,425103646 | 0,031294329 |
| Solyc07g043460.3.1 | GAME6 | -1,359318681 | 1,27E-08 | -0,190700908 | 0,376037687 |
| Solyc07g062520.3.1 | GAME7 | -1,662185084 | 1,26E-14 | -0,636557794 | 0,001151219 |
| Solyc07g043490.1.1 | GAME1 | -0,884034983 | 1,32E-05 | -0,210908043 | 0,231842813 |
| Solyc07g043410.1.1 | GAME2 | 0 | NA | -1,390168387 | 0,745721125 |
| Solyc07g043480.1.1 | GAME17 | -1,03607763 | 1,69E-07 | 0,003807049 | 0,982918559 |
| Solyc07g043500.1.1 | GAME18 | -1,283048358 | 6,02E-06 | -0,24149352 | 0,326668836 |
| Solyc01g090340.2.1 | GAME9 | -1,238433425 | 1,43E-06 | -0,305221634 | 0,188539735 |

**Table S7.** Key genes involved in the biosynthesis of shikimate, chorismate and phenolic compounds in tomato plants. LS-Leaves from *S. indica*-inoculated plants, LC-Leaves from control plants, RS-Roots from *S. indica*-inoculated plants, RC-Roots from control plants, Padj represents the FDR corrected *P*-value [49,50].

| geneID | Annotation | log2FoldChange (LSvsLC) | Padj (LSvsLC) | log2FoldChange (RSvsRC) | Padj (RSvsRC) |
|---|---|---|---|---|---|
| **Shikimate and chorismate** | | | | | |
| Solyc04g074480.3.1 | DAHPS1 | -0,091029449 | 0,518884286 | -0,055184127 | 0,909055509 |
| Solyc11g009080.2.1 | DAHPS2 | -0,448701577 | 6,02E-06 | -0,033685079 | 0,958259072 |
| Solyc01g067750.3.1 | SDH | 0,012339708 | 0,961687305 | 0,11714096 | 0,564414714 |
| Solyc02g094420.3.1 | SK | -0,129120503 | 0,288794119 | 0,030562822 | 0,966040571 |
| Solyc05g050980.3.1 | EPSPS2 | -0,44055303 | 9,06E-05 | -0,034775088 | 0,963068652 |
| Solyc11g017240.2.1 | CM | -0,03445038 | 0,8897592 | 0,100160649 | 0,82565885 |
| Solyc11g066890.1.1 | PAT | -0,332400037 | 0,034328115 | -0,094208126 | 0,87374324 |
| Solyc06g074530.1.1 | PDH | -0,566282705 | 0,040209421 | 0,000422606 | 1 |
| Solyc02g080620.3.1 | PDT | -0,024694318 | 0,949242727 | 0,108618928 | 0,851384887 |
| **Phenylpropanoid** | | | | | |
| Solyc03g042560.2.1 | PAL1 | -0,898417661 | NA | -0,438509133 | NA |
| Solyc00g282510.2.1 | PAL2 | -0,89928451 | NA | 1,247862167 | NA |
| Solyc03g036470.2.1 | PAL3 | -0,171473141 | NA | -0,276371585 | NA |
| Solyc03g071857.1.1 | PAL4 | 0 | NA | -0,000240988 | NA |
| Solyc03g078270.1.1 | PAL5 | NA | NA | NA | NA |
| Solyc09g007890.1.1 | PAL6 | -1,174676907 | 0,479735953 | -1,499501221 | 0,002410206 |
| Solyc09g007900.3.1 | PAL7a | -0,572381395 | 0,015814736 | -0,269295136 | 0,662974208 |
| Solyc09g007910.3.1 | PAL7b | -0,914072434 | 5,03E-05 | -0,256718449 | 0,692940185 |
| Solyc09g007920.3.1 | PAL7c | -0,567828609 | 0,018834789 | -0,277928285 | 0,655692121 |
| Solyc03g071870.1.1 | PAL8 | NA | NA | NA | NA |
| Solyc10g086180.2.1 | PAL9 | -0,736939955 | 6,76E-09 | -0,066904207 | 0,917584149 |
| Solyc05g056170.3.1 | PAL10 | -0,412712138 | 0,019743476 | -0,080605723 | 0,91400179 |
| Solyc01g096670.3.1 | C4H1 | -0,469518807 | 0,001664968 | 0,031852103 | 0,966552457 |
| Solyc06g082530.2.1 | C4H2 | -0,777383791 | 1,72E-06 | -0,006173548 | 1 |
| Solyc03g117870.3.1 | 4CL1 | -0,993874582 | 2,53E-13 | -0,122354545 | 0,805956778 |
| Solyc12g094520.2.1 | 4CL2 | -0,432336753 | 1,93E-06 | -0,161477051 | 0,323557117 |
| Solyc03g097030.3.1 | 4CL3 | -0,71530824 | 6,53E-05 | -0,488191598 | 0,146135686 |
| Solyc06g035960.3.1 | 4CL-l | -0,780536116 | 4,33E-06 | 0,140907509 | 0,838552808 |

**Table S8.** Key genes involved in the biosynthesis of flavonoids, flavonols and anthocyanins in tomato plants. LS-Leaves from *S. indica*-inoculated plants, LC-Leaves from control plants, RS-Roots from *S. indica*-inoculated plants, RC-Roots from control plants, Padj represents the FDR corrected *P*-value [49,50].

| geneID | Annotation | log2FoldChange (LSvsLC) | Padj (LSvsLC) | log2FoldChange (RSvsRC) | Padj (RSvsRC) |
|---|---|---|---|---|---|
| **Flavonoids** | | | | | |
| Solyc09g091510.3.1 | CHS1 | -1,405578312 | 0,005423215 | -1,83876567 | 0,005020187 |
| Solyc05g053550.3.1 | CHS2 | -0,644782121 | 0,240006038 | -0,878582371 | 0,278314997 |
| Solyc05g052240.3.1 | CHI2 | -1,176352071 | 0,000760419 | -1,337554599 | 0,002426868 |
| Solyc02g067870.3.1 | CHI3 | 0,555993317 | 0,869267866 | 0,033847335 | 0,999928216 |
| Solyc05g010320.3.1 | CHI4 | -0,702023761 | 0,00059892 | -0,245995962 | 0,732607255 |
| Solyc07g062030.3.1 | CHI-l | 0,206988033 | 0,132773495 | 0,004272026 | 1 |
| Solyc02g083860.3.1 | F3H | -0,923618013 | 0,000303146 | -0,25221463 | 0,766261324 |
| Solyc03g115220.3.1 | F3'H | -0,758730163 | 0,001524057 | 0,305527499 | 0,514574674 |
| Solyc12g088460.2.1 | F3'H-l | 0,338468515 | 0,208006969 | 0,051909337 | 0,96398595 |
| Solyc11g066580.2.1 | F3'5'H | -0,578543132 | 0,133716272 | -1,267910327 | 0,004625226 |
| **Flavonols** | | | | | |
| Solyc11g013110.2.1 | FLS | -0,8996357 | 1,21E-05 | -0,591266349 | 0,033829586 |
| Solyc10g083440.1.1 | F-3GT | -1,403958107 | 0,001536325 | -1,966460645 | 0,000440105 |
| Solyc12g088170.2.1 | AAC | -0,889554061 | 0,059219576 | -1,148257992 | 0,064127679 |
| Solyc12g098590.2.1 | 5GT | -0,808655047 | 0,097344243 | -0,712729268 | 0,443220755 |
| Solyc09g059170.2.1 | AN-3GT | -1,202746908 | 0,016325381 | -1,486435009 | 0,056143105 |
| Solyc07g055930.3.1 | UGT | -0,778887335 | 0,000130513 | -0,142167332 | 0,860014725 |
| Solyc01g107780.3.1 | Q-3GT | -0,515763727 | 0,308559864 | 0,100130854 | 0,916793324 |
| Solyc02g081690.1.1 | Q-3/4GT | 0,287488471 | 0,404663468 | 0,442879179 | 0,108376132 |
| Solyc11g010760.1.1 | UDP-GT2 | -0,413139373 | 0,01571682 | -0,117639229 | 0,85390753 |
| Solyc03g078770.3.1 | UDP-GT3 | -2,89362296 | 0,04866842 | -0,294369572 | 0,707628688 |
| Solyc05g053890.2.1 | UDP-GTl | -0,567965245 | 0,018152446 | -0,033371818 | 0,986118777 |
| **Anthocyanins** | | | | | |
| Solyc02g085020.3.1 | DFR1 | -0,723238213 | 0,178652891 | -1,145239145 | 0,118180441 |
| Solyc02g089770.3.1 | DFR2 | -1,456494883 | 0,322529114 | 8,37E-05 | 1 |
| Solyc08g080040.3.1 | ANS1 | -0,807279345 | 0,026528347 | 0,669773387 | 0,669849519 |
| Solyc10g076660.2.1 | ANS2 | -3,459172628 | 0,024767673 | 0,669773387 | 0,669849519 |
| Solyc09g059170.2.1 | RT | -1,202746908 | 0,016325381 | -1,486435009 | 0,056143105 |

**Table S9.** Key genes involved in the biosynthesis of alignin and hydroxycinnamic acids in tomato plants. LS-Leaves from *S. indica*-inoculated plants, LC-Leaves from control plants, RS-Roots from *S. indica*-inoculated plants, RC-Roots from control plants, Padj represents the FDR corrected *P*-value [50,88,89].

| geneID | Annotation | log2FoldChange (LSvsLC) | Padj (LSvsLC) | log2FoldChange (RSvsRC) | Padj (RSvsRC) |
|---|---|---|---|---|---|
| **Lignin & HCAs** | | | | | |
| Solyc03g117600.3.1 | HCT | -0,570444609 | 4,23E-08 | -0,223811502 | 0,138655697 |
| Solyc07g005760.3.1 | HQT | -0,084688684 | 0,707674551 | -0,084688684 | 0,707674551 |
| Solyc11g066670.1.1 | UDP-GT1 | -1,12031455 | 5,69E-07 | -0,335791729 | 0,522919538 |
| Solyc02g093250.3.1 | CCoAOMT | -0,709810179 | 0,00967726 | 0,097779953 | 0,925918254 |
| Solyc06g068440.3.1 | CCR1 | -0,815171597 | 6,71E-05 | -0,329673042 | 0,372474776 |
| Solyc03g116910.3.1 | CCR2 | -0,909926964 | 0,014113812 | 0,15246435 | 0,934905037 |
| Solyc08g076780.2.1 | CCR3 | NA | NA | NA | NA |
| Solyc08g005120.3.1 | CCR-l2 | 1,156093528 | 0,479227376 | -0,098499186 | 0,973114754 |
| Solyc06g061280.3.1 | CCR-l4 | 0,961251828 | 0,132557876 | -0,485353696 | 0,601207497 |
| Solyc11g066670.1.1 | UDP-GT1 | -1,12031455 | 5,69E-07 | -0,335791729 | 0,522919538 |
| Solyc02g084570.3.1 | F5H | -0,484482025 | 0,033196492 | -0,227566525 | 0,728433888 |
| Solyc01g107590.3.1 | CAD | -0,468227374 | 4,14E-05 | -0,290902107 | 0,062343787 |
| Solyc01g087630.2.1 | pCAD | -1,076464456 | 0,044052911 | -0,312590108 | 0,882116022 |
| Solyc02g069250.3.1 | pCAD | -1,318336311 | 0,003192601 | 0,993977299 | 0,019930332 |
| Solyc03g078430.2.1 | pCAD | 0 | NA | -0,785863759 | NA |
| Solyc03g078433.1.1 | pCAD | NA | NA | NA | NA |
| Solyc03g078437.1.1 | pCAD | NA | NA | NA | NA |
| Solyc04g048970.1.1 | pCAD | NA | NA | NA | NA |
| Solyc08g014360.2.1 | pCAD | -0,898462445 | NA | 0 | NA |
| Solyc08g048300.2.1 | pCAD | -0,898462445 | NA | 0 | NA |
| Solyc12g055820.2.1 | pCAD | -0,071222154 | 0,611534601 | -0,00820372 | 0,996038378 |
| Solyc03g080180.3.1 | COMT | -0,914019261 | 2,01E-11 | -0,35010867 | 0,068918999 |
| Solyc10g078240.2.1 | SlC3H4 | -1,004826422 | 2,01E-11 | -0,201064207 | 0,635129215 |
| Solyc01g096670.3.1 | SlC3H3 | -0,469518807 | 0,001664968 | 0,031852103 | 0,966552457 |
| Solyc10g078220.2.1 | SlC3H1 | 3,230724147 | 0,008537938 | 3,638829219 | 0,029683717 |
| Solyc10g078230.2.1 | SlC3H2 | 1,539907 | 0,084764268 | 1,308337238 | 0,345907924 |
| Solyc04g010250.3.1 | pCSE | 1,223489703 | 9,48E-15 | 0,639263733 | 4,02E-05 |
| Solyc07g052510.4.1 | TPX1 | 1,594725994 | 0,069343572 | 0,133663786 | 0,812490037 |
| Solyc07g017880.3.1 | POD | 0 | 1 | 0,239891571 | 0,67872875 |
| Solyc02g084790.3.1 | POD | -0,163331214 | 0,983201705 | 0,467952915 | 0,372584118 |
| Solyc10g076240.2.1 | POD | 0 | 1 | 0,58482129 | 0,026963089 |
| Solyc03g006700.3.1 | POD | 0,329869405 | 0,31167089 | -0,120926361 | 0,804816973 |
| Solyc02g079500.4.1 | POD | 0,78714786 | 0,009665615 | 0,875455523 | 2,36E-06 |
| Solyc04g071890.3.1 | POD | 0,829452925 | 0,001563457 | 0,18776353 | 0,845572452 |

**Table S10.** Putative fatty acid desaturases expressed in leaves and roots in tomato plants. LS-Leaves from *S. indica*-inoculated plants, LC-Leaves from control plants, RS-Roots from *S. indica*-inoculated plants, RC-Roots from control plants, Padj represents the FDR corrected *P*-value**.**

| geneID | Annotation | log2FoldChange (LSvsLC) | Padj (LSvsLC) | log2FoldChange (RSvsRC) | Padj (RSvsRC) |
|---|---|---|---|---|---|
| Solyc04g040130.1.1 | | 3,198652 | 0,004083 | 2,294026 | 2,63E-08 |
| Solyc12g044950.2.1 | | 0,897417 | 0,019481 | 1,452144 | 5,23E-05 |
| Solyc12g100240.1.1 | | 0 | 1 | 1,377983 | 0,112193 |
| Solyc12g100250.2.1 | | 0 | 1 | 3,304992 | 1,42E-05 |
| Solyc12g100260.1.1 | | 0 | 1 | 3,419097 | 3,54E-09 |
| Solyc12g100270.2.1 | | 1,581493 | 0,034177 | 1,736664 | 1,20E-06 |
| Solyc12g049030.1.1 | | 3,757193 | 0,0203 | 4,57847 | 5,09E-05 |
| Solyc10g011810.3.1 | | -0,0151 | 0,965894 | 0,438785 | 0,000647 |
| Solyc01g088400.3.1 | | -0,16255 | 0,297263 | 0,189514 | 0,628794 |
| Solyc01g006430.3.1 | | -0,93606 | 3,26E-17 | 0,235283 | 0,24383 |
| Solyc03g063110.3.1 | | -0,04009 | 0,872316 | 0,021389 | 0,984577 |
| Solyc04g040120.1.1 | | 0 | 1 | 0,313254 | 0,917584 |
| Solyc05g007460.3.1 | | -0,40774 | 0,004627 | 0,057637 | 0,927733 |
| Solyc08g063090.2.1 | | -0,20588 | 0,590021 | -0,70359 | 0,676064 |
| Solyc11g008680.2.1 | | 0,397876 | 0,183515 | -0,1575 | 0,880243 |
| Solyc07g005510.3.1 | | -0,63079 | 3,74E-07 | -0,18965 | 0,568441 |
| Solyc03g116730.3.1 | | -0,55934 | 0,001785 | -0,22752 | 0,748806 |
| Solyc04g072180.2.1 | | -0,23171 | 0,030744 | 0,104446 | 0,706116 |
| Solyc02g086180.3.1 | | -1,93645 | 2,22E-09 | -0,14203 | 0,937103 |
| Solyc05g050090.3.1 | | -0,63783 | 0,003528 | -0,18808 | 0,879373 |
| Solyc01g091320.3.1 | | -2,18952 | 6,15E-19 | -0,23474 | 0,802902 |
| Solyc01g010860.3.1 | | -0,02066 | 0,957769 | 0,060875 | 0,920814 |
| Solyc06g059710.3.1 | | -0,26197 | 0,218846 | -1,3699 | 0,769268 |
| Solyc05g010180.3.1 | | -0,15995 | 0,526267 | -0,39711 | 0,218499 |
| Solyc08g079570.3.1 | | -0,05894 | 0,69955 | 0,162848 | 0,297973 |
| Solyc03g117800.3.1 | | -0,9662 | 0,032004 | -1,30899 | 0,000807 |
| Solyc03g058430.2.1 | | -0,16184 | 0,348322 | 0,15178 | 0,722384 |
| Solyc06g076410.3.1 | | -0,3262 | 0,000596 | -0,29634 | 0,004736 |
| Solyc01g088420.3.1 | | 0,141716 | 0,950398 | 3,171774 | 0,166854 |
| Solyc06g051400.3.1 | | -0,52122 | 0,000237 | -0,34201 | 0,112681 |
| Solyc06g053480.3.1 | | -0,04135 | 0,822722 | -0,04946 | 0,926497 |
| Solyc01g097810.3.1 | | -0,05769 | 0,70729 | -0,24641 | 0,087337 |
| Solyc02g063240.3.1 | | 0,199096 | 0,148221 | 0,23264 | 0,227459 |
| Solyc07g006300.3.1 | | -0,69192 | 0,003193 | 0,043613 | 0,986856 |
| Solyc02g085250.3.1 | | 0,009023 | 0,990782 | -0,00047 | 1 |
| Solyc10g011820.3.1 | | -0,94553 | 6,04E-09 | 0,109334 | 0,886557 |
| Solyc06g007130.4.1 | | 0,690138 | 7,02E-06 | 0,160779 | 0,747131 |
| Solyc03g123760.3.1 | | 0,064769 | 0,793503 | -0,10239 | 0,87071 |
| Solyc10g081650.2.1 | | -0,04063 | 0,884182 | -0,16263 | 0,691256 |
| Solyc03g065250.3.1 | | 0,417943 | 0,034685 | -0,66939 | 0,019346 |

**Table S11.** Genes involved in the mevalonate (MVA) and MEP pathway in tomato. LS-Leaves from *S. indica*-inoculated plants, LC-Leaves from control plants, RS-Roots from *S. indica*-inoculated plants, RC-Roots from control plants, Padj represents the FDR corrected *P*-value [82].

| **geneID** | **Annotation** | **log2FoldChange (LSvsLC)** | **Padj (LSvsLC)** | **log2FoldChange (RSvsRC)** | **Padj (RSvsRC)** |
|---|---|---|---|---|---|
| Solyc07g045350.3.1 | AACT.ch7 | 0,057950542 | 0,86401304 | 0,061477604 | 0,933604967 |
| Solyc05g017760.3.1 | AACT.ch5 | -0,185388001 | 0,30353754 | -0,009889917 | 0,997108054 |
| Solyc08g080170.3.1 | HMGS.ch8 | -0,042359663 | 0,91085945 | -0,050201796 | 0,961220221 |
| Solyc08g080160.3.1 | HMGS.ch8 | 0,264529744 | NA | -1,98178398 | NA |
| Solyc12g056450.2.1 | HMGS.ch12 | -0,185305786 | 0,13557104 | -0,101299395 | 0,77041856 |
| Solyc02g082260.3.1 | HMGR.ch2 | -0,41414197 | 0,00625572 | 0,085593114 | 0,891536188 |
| Solyc02g038740.3.1 | HMGR.ch2* | -0,929268138 | 3,73E-05 | 0,109239354 | 0,91996693 |
| Solyc03g032010.3.1 | HMGR1.ch3 | -1,071862292 | 2,71E-12 | 0,103935545 | 0,886556654 |
| Solyc03g032020.3.1 | HMGR.ch3 | -1,006995835 | 4,91E-06 | -0,263899143 | 0,693595505 |
| Solyc01g098840.3.1 | MVK.ch1 | -0,058623672 | 0,65514158 | 0,031626634 | 0,944435225 |
| Solyc06g066310.3.1 | pMVK.ch6 | -0,007319725 | 1 | 0,035535642 | 0,959856798 |
| Solyc08g076140.3.1 | pMVK.ch8 | -0,184895099 | 0,05873937 | 0,045112927 | 0,896239626 |
| Solyc04g009650.3.1 | MDC.ch4 | -0,418461713 | 0,09807263 | 0,132787489 | 0,859066682 |
| Solyc11g007020.2.1 | MDC.ch11 | -0,063861507 | 0,73740772 | 0,017151174 | 0,987660236 |
| Solyc04g056390.3.1 | IDI.ch4 | -0,088246492 | 0,32845503 | 0,012939541 | 0,983146426 |
| Solyc05g055760.3.1 | IDI.ch5 | -0,131862568 | 0,5720865 | 0,08163444 | 0,90972484 |
| Solyc01g067890.3.1 | DXS1.ch1 | -0,551888421 | 0,00566032 | -0,151254629 | 0,846599315 |
| Solyc06g071380.3.1 | DXS.ch6 | -0,191929361 | 0,44383373 | 0,186955286 | 0,77041856 |
| Solyc11g010850.2.1 | DXS2.ch11 | -0,919166826 | 0,0097868 | -0,261579324 | 0,881177812 |
| Solyc03g114340.3.1 | DXR.ch3 | -0,156834036 | 0,16013763 | -0,136228401 | 0,560526059 |
| Solyc01g102820.3.1 | MCT.ch1a | 0,033459324 | 0,85579128 | 0,055696128 | 0,913819537 |
| Solyc01g049890.3.1 | MCT.ch1b | 0,091507575 | 0,19974777 | -0,008736578 | 0,987861872 |
| Solyc01g009010.3.1 | CMK.ch1 | -0,031884314 | 0,84958923 | -0,198349707 | 0,241942195 |
| Solyc08g081570.3.1 | MDS.ch8 | 0,150892116 | 0,16638511 | 0,048971282 | 0,926754359 |
| Solyc11g069380.2.1 | HDS.ch11 | -0,129439941 | 0,30313817 | -0,189595803 | 0,309457903 |
| Solyc01g109300.3.1 | HDR.ch1 | -0,158669455 | 0,44433905 | -0,074151939 | 0,925426569 |

**Table S12.** Genes encoding for *trans*- and *cis*-prenyltransferases involved in precursor biosynthesis of terpenes in tomato, LS-Leaves from *S. indica*-inoculated plants, LC-Leaves from control plants, RS-Roots from *S. indica*-inoculated plants, RC-Roots from control plants, Padj represents the FDR corrected *P*-value [82].

| **geneID** | **Annotation** | **log2FoldChange (LSvsLC)** | **Padj (LSvsLC)** | **log2FoldChange (RSvsRC)** | **Padj (RSvsRC)** |
|---|---|---|---|---|---|
| Solyc08g023470.3.1 | GPS.ch8 | 0,161009402 | 0,08502987 | 0,03684977 | 0,926754359 |
| Solyc12g015860.2.1 | FPS.ch12 | -0,120645626 | 0,3868077 | 0,062953783 | 0,896942232 |
| Solyc10g005820.2.1 | FPS.ch10a | -0,098523742 | 0,78937338 | 0,039549313 | 0,977682299 |
| Solyc10g005840.3.1 | FPS.ch10b | 0,127272475 | 0,37757649 | -0,111808618 | 0,754346618 |
| Solyc04g079960.1.1 | GGPPS.ch4 | 0,056239045 | 0,7208673 | 0,239430733 | 0,435398055 |
| Solyc09g008920.3.1 | GGPPS.ch9 | 0,108136192 | 0,29281316 | 0,007358012 | 0,995739606 |
| Solyc02g085700.1.1 | GGPPS.ch2 | 0,056057941 | 0,68947972 | -0,283364382 | 0,033126615 |
| Solyc08g005680.3.1 | CPT1.ch8 | -0,168138439 | 0,83150306 | 1,703465261 | 0,645906245 |
| Solyc08g005660.2.1 | CPT2.ch8 | 1,493110427 | NA | 0 | NA |
| Solyc03g025560.3.1 | CPT3.ch3 | 0,938931372 | 5,98E-09 | 0,063686629 | 0,931339242 |
| Solyc10g085150.2.1 | CPT4.ch10 | 0,085612234 | 0,7048527 | 0,089889705 | 0,880373283 |
| Solyc10g085140.1.1 | CPT5.ch10 | 0,519904248 | 0,35155701 | -0,512426209 | 0,65582371 |
| Solyc06g059990.3.1 | CPT6.ch6 | 0 | 1 | -0,324919447 | 0,691476966 |
| Solyc06g076920.3.1 | CPT7.ch6 | -0,124522285 | 0,68327154 | 0,271683457 | 0,972197923 |

**Table S13.** Terpene synthase gene family in tomato. LS-Leaves from *S. indica*-inoculated plants, LC-Leaves from control plants, RS-Roots from *S. indica*-inoculated plants, RC-Roots from control plants, Padj represents the FDR corrected *P*-value [54].

| geneID | Annotation | log2FoldChange (LSvsLC) | Padj (LSvsLC) | log2FoldChange (RSvsRC) | Padj (RSvsRC) |
|---|---|---|---|---|---|
| Solyc01g105850.3.1 | TPS1 | -0,54994748 | 0,7208673 | 0 | NA |
| Solyc01g105910.2.1 | TPS2 | NA | NA | NA | NA |
| Solyc01g105870.3.1 | TPS3 | 0,580415632 | NA | 0,035808782 | NA |
| Solyc01g105880.4.1 | TPS4 | 0 | 1 | -0,975821005 | 0,478825032 |
| Solyc01g105890.3.1 | TPS5 | -1,113476057 | 2,02E-07 | 0,62955363 | 0,946881956 |
| Solyc01g105900.2.1 | TPS6 | NA | NA | NA | NA |
| Solyc01g105920.3.1 | TPS7 | -0,703745041 | 0,16072763 | -0,634129923 | 0,852887879 |
| Solyc01g105960.2.1 | TPS8 | -1,64198066 | 0,46879539 | -0,174702613 | 0,911475706 |
| Solyc06g059885.1.1 | TPS9 | -0,405747158 | 0,03477536 | -0,059272384 | 1 |
| Solyc06g059930.3.1 | TPS12 | -0,418507469 | 0,28653169 | 0 | 1 |
| Solyc09g092470.2.1 | TPS14 | -0,924272171 | 0,73448105 | -0,172113512 | 0,844577631 |
| Solyc07g051940.3.1 | TPS15 | 0,215918118 | 0,86035072 | -0,062621214 | 0,999809093 |
| Solyc07g008690.3.1 | TPS16 | 0,088974328 | 0,9809211 | 0 | 1 |
| Solyc12g006570.2.1 | TPS17 | 0,583108512 | 0,7444946 | -0,508364553 | NA |
| Solyc08g005720.3.1 | TPS18 | -0,441017097 | 0,34874294 | -0,047332024 | 0,971318204 |
| Solyc08g005665.1.1 | TPS20 | 0,136657211 | 0,76722836 | 3,768573001 | 0,025929723 |
| Solyc08g005640.3.1 | TPS21 | -0,213635236 | 0,66685141 | -0,315605501 | 0,826897157 |
| Solyc07g066670.3.1 | TPS24 | 0,078842979 | 0,71287643 | -0,106766107 | 0,809565188 |
| Solyc02g079890.2.1 | TPS25 | -1,336823602 | NA | 0 | NA |
| Solyc02g079910.2.1 | TPS27 | -2,682785575 | NA | 0 | NA |
| Solyc04g054380.1.1 | TPS28 | NA | NA | NA | NA |
| Solyc12g019240.2.1 | TPS30 | NA | NA | NA | NA |
| Solyc01g101170.3.1 | TPS31 | -0,035906624 | 0,99941245 | 1,214155049 | 0,091506913 |
| Solyc01g101180.3.1 | TPS32 | -0,359959576 | NA | 4,732970263 | NA |
| Solyc01g101190.3.1 | TPS33 | 0,596821351 | 0,71653315 | 5,113598894 | NA |
| Solyc01g101210.4.1 | TPS35 | -1,452780804 | NA | -0,611150631 | NA |
| Solyc06g060180.2.1 | TPS36 | NA | NA | NA | NA |
| Solyc10g005410.3.1 | TPS38 | -0,515268314 | 0,56814557 | -0,032229788 | 1 |
| Solyc02g079840.2.1 | TPS39 | 0,765313276 | 0,75277623 | 0,091578319 | 0,99808769 |
| Solyc06g084240.2.1 | TPS40 | -1,286341356 | 0,2674703 | -1,052006644 | NA |
| Solyc08g005710.3.1 | TPS41 | -0,226917392 | 0,54397277 | 0,495493666 | 0,81218199 |
| Solyc10g005420.1.1 | TPS42 | NA | NA | NA | NA |
| Solyc03g006550.3.1 | TPS46 | -0,603841912 | 0,65298683 | 0 | NA |
| Solyc03g007730.1.1 | TPS47 | 0,569118998 | NA | 0,631877319 | NA |
| Solyc04g051620.2.1 | TPS48 | 0 | NA | -1,645031122 | NA |
| Solyc07g052120.3.1 | TPS51 | 2,849151536 | 0,045501 | 0,797192056 | 0,532046929 |

**Table S14.** Transcription factors considered to be involved in terpene biosynthesis in tomato. LS-Leaves from *S. indica*-inoculated plants, LC-Leaves from control plants, RS-Roots from *S. indica*-inoculated plants, RC-Roots from control plants, Padj represents the FDR corrected *P*-value [82].

| **geneID** | **Annotation** | **log2FoldChange (LSvsLC)** | **Padj (LSvsLC)** | **log2FoldChange (RSvsRC)** | **Padj (RSvsRC)** |
|---|---|---|---|---|---|
| Solyc05g052050.1.1 | SlAP2_9 | 0,339444074 | 0,10726132 | 0,212137882 | 0,70131784 |
| Solyc11g072600.2.1 | SlAP2_6 | 0,069385254 | 0,7541838 | -0,028227528 | 0,977664088 |
| Solyc10g050970.1.1 | SlAP2_4 | -1,702544451 | NA | -2,911016156 | NA |
| Solyc06g075510.3.1 | SlAP2_10 | -0,246252057 | 0,10915844 | -0,189316011 | 0,515283776 |
| Solyc12g056590.2.1 | SlAP2_5 | 0,311043086 | 0,58918874 | 0,329584578 | 0,323332603 |
| Solyc09g075420.3.1 | SlAP2_11 | 0,794996508 | 0,00057209 | 0,203805019 | 0,806174798 |
| Solyc05g050830.2.1 | SlAP2_7 | -1,231894948 | 0,00247871 | -2,755635583 | 0,215330769 |
| Solyc03g026270.3.1 | SlAP2_2 | -3,028177242 | 0,1287803 | -4,641461137 | 0,094800438 |
| Solyc01g108240.3.1 | SlAP2_1 | -1,235683192 | 0,6562689 | -5,205940302 | NA |
| Solyc07g053740.1.1 | SlAP2_8 | -1,44036617 | 0,00682981 | -0,378952572 | 0,857937386 |
| Solyc01g095100.3.1 | SlWRKY22 | -0,765603305 | 0,00640675 | 0,06312119 | 0,97055289 |
| Solyc12g011200.2.1 | SlWRKY28 | -0,268752572 | 0,68442332 | 0,346166031 | 0,65582371 |
| Solyc03g113120.3.1 | SlWRKY73 | -0,590197796 | 0,42635358 | 0,417725729 | 0,572987269 |
| Solyc07g055280.3.1 | SlWRKY78 | 0,380688852 | 0,1476444 | 0,078348944 | 0,945974768 |
| Solyc08g005050.3.1 | SlMYC1 | -0,558751919 | 0,00177769 | -0,477686159 | 0,03402615 |
| Solyc02g062400.3.1 | EOT1 | -0,522319521 | 0,20536434 | -0,77095575 | 0,926770625 |
| Solyc00g117450.2.1 | EOT2 | -0,850562368 | 0,38547123 | 0,551094685 | 0,911357047 |
| Solyc02g084680.2.1 | EOT3 | -0,319305473 | 0,44288983 | 0,551387136 | 0,457772107 |

**Table S15.** Primer list

| No | Name | Sequence 5′-3′ | Used for |
|---|---|---|---|
| **1** | SlTPS5Q-F | CTTCGGATGAACTGAAAAGAGG | qRT-PCR |
| **2** | SlTPS5Q-R | GTGGAGAATTTTTGCTTTGAGC | qRT-PCR |
| **3** | SlTPS12Q-F | CGTTACTGGTTGAGTCTGTCG | qRT-PCR |
| **4** | SlTPS12Q-R | CTTGATTATTTGAAATATTCCGGAGG | qRT-PCR |
| **5** | SlTPS20Q-F | AAGGAAATCTTGGAAATGAATAGAA | qRT-PCR |
| **6** | SlTPS20Q-R | ATAGAAGGAAAGAACAAAAGTCATAA | qRT-PCR |
| **7** | SlTPS47Q-F | CTTGAAAGACGTCATCTCTATGGTG | qRT-PCR |
| **8** | SlTPS47Q-R | CTGACACAAAGATAAAACAACATCAAG | qRT-PCR |
| **9** | SlTPS47_USER-F | GGCTTAAUATGGTCGTTGGATTTGG | USER® cloning for *N. benthamiana* expression |
| **10** | SlTPS47_USER-R | GGTTTAAUTCATGCTTTTATAGGATC | USER® cloning for *N. benthamiana* expression |

**Table S16.** Key genes involved in jasmonic acid (JA) biosynthesis and signalling in tomato plants. LS-Leaves from *S. indica*-inoculated plants, LC-Leaves from control plants, RS-Roots from *S. indica*-inoculated plants, RC-Roots from control plants, Padj represents the FDR corrected P-value [90]**.**

| geneID | Annotation | log2FoldChange (LSvsLC) | Padj (LSvsLC) | log2FoldChange (RSvsRC) | Padj (RSvsRC) |
|---|---|---|---|---|---|
| Solyc06g051400.3.1 | FAD7 | -0,52122 | 0,000237 | -0,34201 | 0,112681 |
| Solyc06g007130.4.1 | FAD3 | 0,690138 | 7,02E-06 | 0,160779 | 0,747131 |
| Solyc02g077110.3.1 | LPS1 | -0,88486 | 0,013511 | -0,59014 | 0,533914 |
| Solyc02g077420.3.1 | LPS2 | 0,208364 | 0,64 | -0,83081 | 0,016525 |
| Solyc02g077430.3.1 | LPS3 | 0,383799 | 0,153999 | -0,54053 | 0,098868 |
| Solyc08g014000.3.1 | LOXa | 0,371724 | 0,665065 | -0,24131 | 0,448477 |
| Solyc01g099190.3.1 | LOXb | 1,876023 | 0,388878 | -0,98646 | NA |
| Solyc01g006540.3.1 | LOXc | -0,01829 | 0,973535 | -1,43732 | 0,426245 |
| Solyc03g122340.3.1 | LOXd | -1,0794 | 3,56E-08 | -0,21732 | 0,740042 |
| Solyc04g079730.1.1 | AOS1 | -0,36596 | 0,669424 | -0,23212 | 0,916888 |
| Solyc11g069800.1.1 | AOS2 | 0,237965 | 0,148081 | 0,294209 | 0,160026 |
| Solyc10g007960.1.1 | AOS3 | 0 | 1 | 0,264679 | 0,920446 |
| Solyc02g085730.3.1 | AOC | -0,56523 | 0,001218 | -0,06737 | 0,943924 |
| Solyc07g007870.3.1 | OPR3 | -0,213 | 0,064294 | -0,16317 | 0,39408 |
| Solyc01g103390.3.1 | OPR2 | -0,1053 | 0,821924 | 0,492251 | 0,122428 |
| Solyc08g078390.3.1 | ACX1a | -0,0428 | 0,673255 | -0,10809 | 0,35879 |
| Solyc10g009600.1.1 | SlJAR1 | 0 | NA | -0,8944 | NA |
| Solyc10g011660.3.1 | SlJRL1 | 0,042699 | 0,87078 | -0,05252 | 0,941463 |
| Solyc05g052620.3.1 | COI1 | -0,1008 | 0,457458 | 0,079088 | 0,839469 |
| Solyc07g042170.3.1 | JAZ1 | 0,052028 | 0,832774 | -0,37273 | 0,007447 |
| Solyc12g009220.2.1 | JAZ2 | 0,271821 | 0,678566 | -0,38681 | 0,821226 |
| Solyc03g122190.3.1 | JAZ3 | -0,39368 | 0,0003 | -0,09444 | 0,911305 |
| Solyc12g049400.2.1 | JAZ4 | 0,140274 | 0,937416 | 0,54339 | 0,890906 |
| Solyc03g118500.3.1 | JAZ5 | -0,01954 | 0,941306 | -0,00302 | 1 |
| Solyc01g005440.3.1 | JAZ6 | 0,012189 | 0,984196 | 0,114442 | 0,812334 |
| Solyc11g011030.2.1 | JAZ7 | 0,077566 | 0,908856 | -0,75411 | 0,612208 |
| Solyc06g068930.2.1 | JAZ8 | 0 | 1 | -0,26571 | 0,574543 |
| Solyc08g036640.3.1 | JAZ9 | 0,541241 | NA | -0,90516 | NA |

| | | | | | |
|---|---|---|---|---|---|
| Solyc08g036620.3.1 | JAZ10 | -0,89846 | NA | 0 | NA |
| Solyc08g036660.3.1 | JAZ11 | -0,19585 | NA | 0 | NA |
| Solyc01g009740.2.1 | JAZ12 | NA | NA | NA | NA |
| Solyc08g076930.1.1 | MYC2 | -0,49978 | 0,00035 | -0,17132 | 0,649645 |
| Solyc08g005050.3.1 | MYC10 | -0,55875 | 0,001778 | -0,47769 | 0,034026 |
| Solyc00g071180.3.1 | CysPI | 2,905347 | 0,055295 | -0,31417 | 0,892171 |
| Solyc08g074630.2.1 | PPO F | -1,04885 | 0,065938 | 0,662129 | 0,945518 |
| Solyc08g074620.3.1 | PPO F | 0,046456 | 0,948825 | 0,651306 | 0,944637 |
| Solyc08g074680.3.1 | PPO B | -0,90066 | 0,740395 | -0,18542 | 0,709324 |
| Solyc03g098790.3.1 | CDI | -0,78679 | NA | 0 | NA |
| Solyc09g008670.3.1 | TD2 | 0,215593 | 0,889864 | -1,29753 | 0,226864 |
| Solyc12g010020.2.1 | LapA1 | -0,07866 | 0,986593 | 0 | 1 |
| Solyc00g187050.3.1 | LapA2 | 0 | NA | 0,656818 | NA |
| Solyc01g091160.4.1 | LeARG1 | 0,208857 | 0,524094 | -0,08599 | 0,943413 |
| Solyc01g091170.3.1 | LeARG2 | 0,568832 | 0,463449 | 0 | 1 |
| Solyc09g084470.3.1 | PI-I | -0,98887 | 0,19942 | 0 | 1 |
| Solyc03g020080.3.1 | PI-II | NA | NA | NA | NA |
| Solyc09g091540.1.1 | SAMT | NA | NA | NA | NA |

**Table S17.** Key genes involved in salicylic acid (SA) biosynthesis and signalling in tomato plants. LS-Leaves from *S. indica*-inoculated plants, LC-Leaves from control plants, RS-Roots from *S. indica*-inoculated plants, RC-Roots from control plants, Padj represents the FDR corrected *P*-value [90].

| geneID | Annotation | log2FoldChange (LSvsLC) | Padj (LSvsLC) | log2FoldChange (RSvsRC) | Padj (RSvsRC) |
|---|---|---|---|---|---|
| Solyc03g042560.2.1 | PAL1 | -0,89842 | NA | -0,43851 | NA |
| Solyc00g282510.2.1 | PAL2 | -0,89928 | NA | 1,247862 | NA |
| Solyc03g036470.2.1 | PAL3 | -0,17147 | NA | -0,27637 | NA |
| Solyc03g071857.1.1 | PAL4 | 0 | NA | -0,00024 | NA |
| Solyc03g078270.1.1 | PAL5 | NA | NA | NA | NA |
| Solyc09g007890.1.1 | PAL6 | -1,17468 | 0,479736 | -1,4995 | 0,00241 |
| Solyc09g007900.3.1 | PAL7a | -0,57238 | 0,015815 | -0,2693 | 0,662974 |
| Solyc09g007910.3.1 | PAL7b | -0,91407 | 5,03E-05 | -0,25672 | 0,69294 |
| Solyc09g007920.3.1 | PAL7c | -0,56783 | 0,018835 | -0,27793 | 0,655692 |
| Solyc03g071870.1.1 | PAL8 | NA | NA | NA | NA |
| Solyc10g086180.2.1 | PAL9 | -0,73694 | 6,76E-09 | -0,0669 | 0,917584 |
| Solyc05g056170.3.1 | PAL10 | -0,41271 | 0,019743 | -0,08061 | 0,914002 |
| Solyc06g071030.3.1 | ICS | -0,64177 | 1,03E-06 | -0,21447 | 0,775642 |
| Solyc07g040690.3.1 | NPR1 | -0,51342 | 2,99E-05 | -0,06393 | 0,909451 |
| Solyc04g011670.3.1 | TGA1a | 0,123371 | 0,588393 | 0,005003 | 1 |
| Solyc10g080770.2.1 | TGA2.1. | -0,68935 | 8,16E-08 | 0,130224 | 0,766083 |
| Solyc11g064950.2.1 | TGA2.2. | -0,15307 | 0,444841 | 0,055487 | 0,937103 |
| Solyc01g095630.3.1 | WRKY70 | -0,37616 | 0,317893 | -0,29719 | 0,775505 |
| Solyc01g008620.3.1 | β-1,3-glucanase | 3,278558 | 3,16E-11 | 3,464475 | 1,83E-09 |
| Solyc08g080620.1.1 | PR-5 | -1,85628 | NA | -0,49681 | NA |
| Solyc01g097240.3.1 | PR-2 | 0,815339 | 0,132863 | 4,411324 | 1,71E-18 |
| Solyc09g007010.1.1 | PR-1 | 0 | NA | 1,954777 | NA |
| Solyc08g079870.3.1 | Subtilisin | 0,676755 | 0,584392 | 4,560762 | 0,000319 |
| Solyc08g079840.2.1 | Subtilisin | 0,46431 | 0,000379 | 0,33405 | 0,763955 |
| Solyc02g082920.3.1 | Chi2 | 0,239727 | 0,655921 | 1,961853 | 2,21E-08 |
| Solyc05g050130.3.1 | acidic chitinase | 0,261984 | 0,561059 | 0,648983 | 0,1249 |
| Solyc09g090990.2.1 | PRP-like | 0,576715 | 0,856787 | 1,319672 | 3,11E-05 |
| Solyc08g080650.3.1 | Osmotin-like | 0,867424 | 0,003283 | 0,392173 | 0,54484 |
| Solyc04g049090.3.1 | MLO-like | 0,490008 | 0,000686 | 0,413785 | 0,028852 |
| Solyc01g006350.3.1 | glucan synthase | -0,08513 | 0,303008 | 0,065758 | 0,752887 |
| Solyc02g069310.3.1 | Nim | -1,14235 | 6,48E-16 | 0,003057 | 1 |
| Solyc10g011910.3.1 | WRKY22 | -0,25548 | 0,715676 | -0,26804 | 0,852181 |
| Solyc01g095100.3.1 | WRKY22.1 | -0,7656 | 0,006407 | 0,063121 | 0,970553 |

## Supplementary Figures

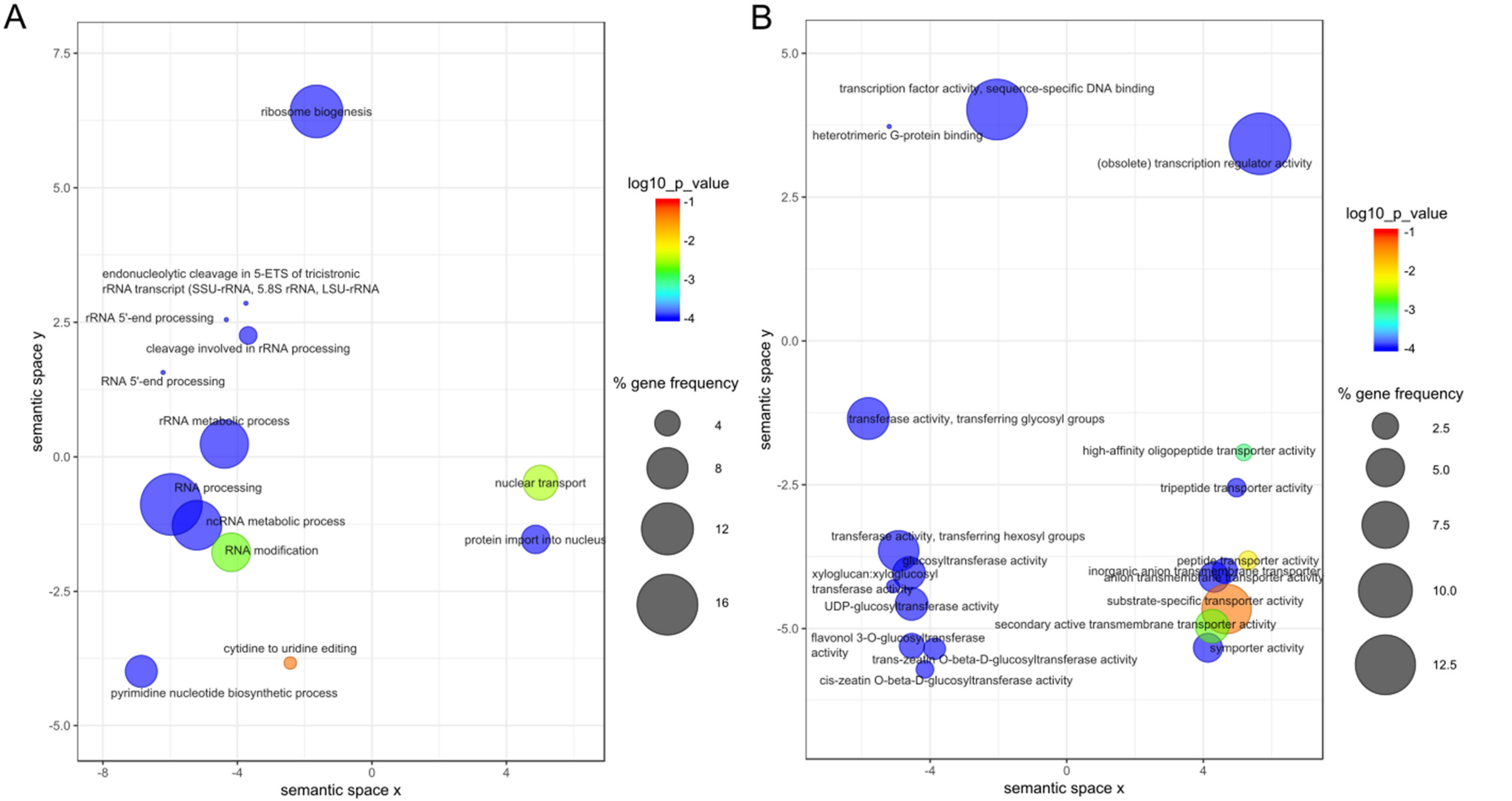


**Figure S1.** REVIGO scatter plot showing the selected GO terms enriched (FDR corrected *P* ≤0.05) in the significantly **A)** up-regulated and **B)** down-regulated genes in leaves of *S. indica*-colonised plants. Cluster representatives are presented here (i.e. terms remaining after the redundancy reduction) in a two dimensional space derived by applying multidimensional scaling to a matrix of the GO terms' semantic similarities. Colour range is showing the $Log_{10}$ of the simulation corrected *P*-value of enrichment and the size of the bubbles reflects the percentage of gene frequency (The percentage of genes involved in the GO category in the total number of significantly up-regulated genes).

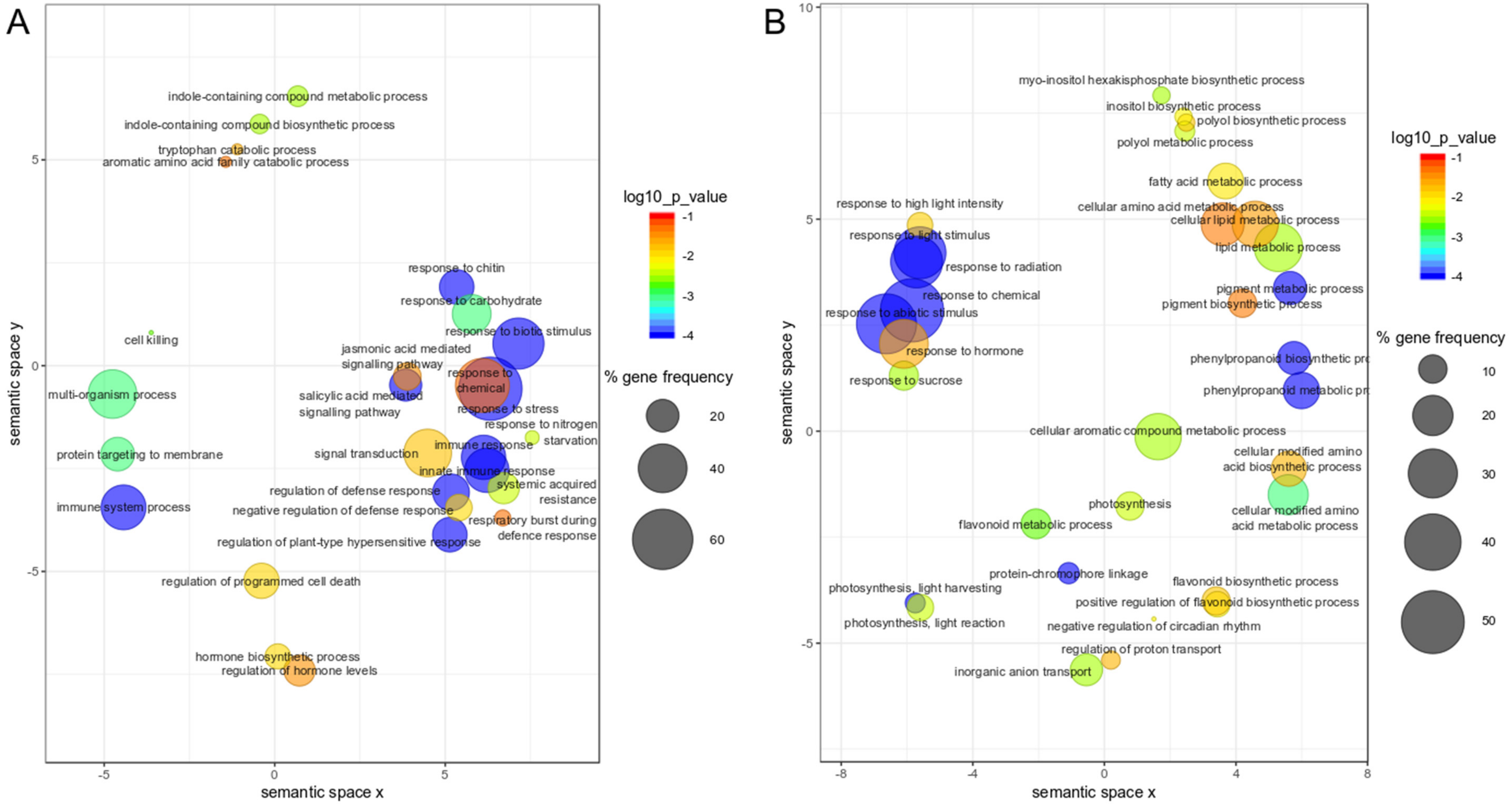


**Figure S2.** REVIGO scatter plot showing selected GO terms enriched (simulation corrected P ≤ 0.05) in the significantly A) up-regulated and B) down-regulated genes in roots colonised by *S. indica*. Cluster representatives are presented here (i.e. terms remaining after the redundancy reduction) in a two-dimensional space derived by applying multidimensional scaling to a matrix of the GO terms' semantic similarities. Colour range is showing the Log10 of the simulation corrected P-value of enrichment and the size of the bubbles reflects the percentage of gene frequency (the percentage of genes involved in the GO category to the total number of significantly two-fold up-regulated genes).

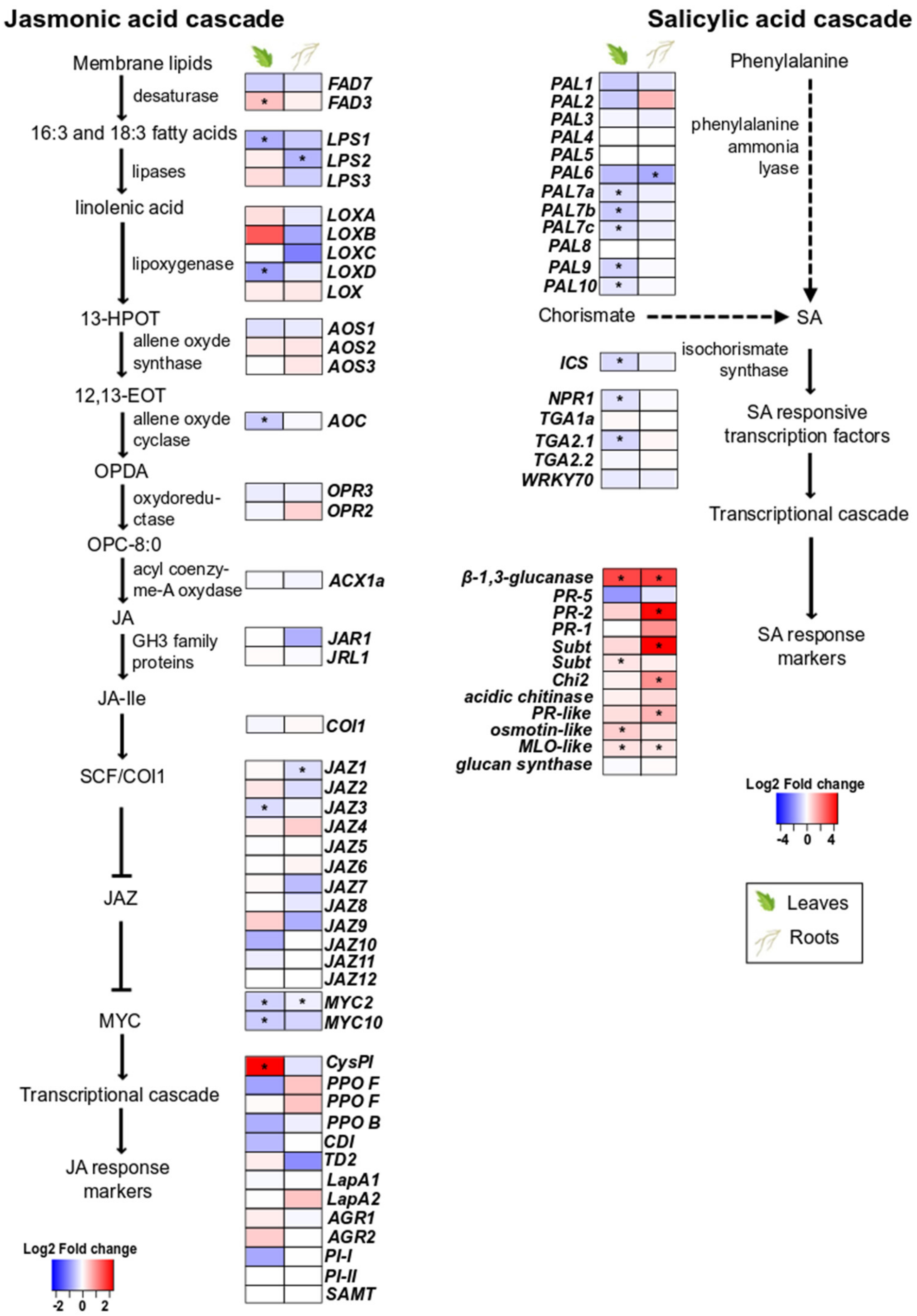


**Figure S3.** Effect of *S. indica*-colonisation on the expression of genes involved in the JA and SA biosynthesis and signalling pathways. Changes in gene expression between tissues from inoculated plants and tissues from endophyte-free plants are represented as the $Log_2$ of the fold change in the form of a heatmap. Data derived from RNA sequencing on leaves and roots form *S. indica*- and endophyte-free plants. Asterisks (*) indicate FDR corrected $P \leq 0.05$ (transformed from [90].

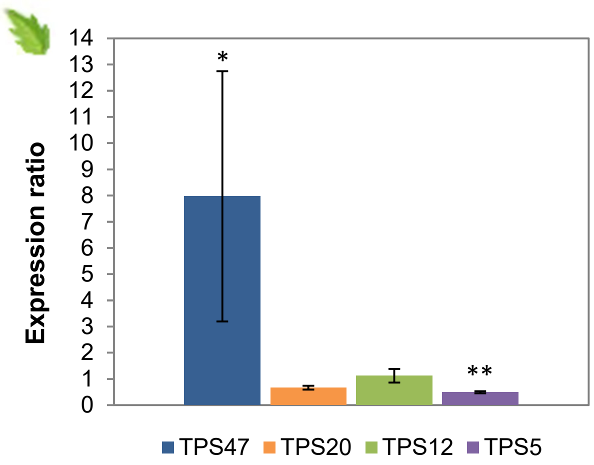


**Figure S4.** Expression ratio of tomato *TPS*s in leaves of colonised compared to leaves of endophyte-free plants. Data obtained using RT-qPCR on leaf RNA. Normalisation of expression data was done using expression level of *SlTUB* (GenBank: DQ205342.1) expression levels. Calculation of gene expression ratio and statistical analyses were performed using the software tool REST© [91]. Error bars represent the standard deviation of the mean (*$P \leq 0.05$, **$P \leq 0.005$).

## Supplementary Methods

## Validation of tomato TPS expression by RT-qPCR

The RNA samples sent for sequencing were used to synthesize cDNA and validate the expression of tomato *TPS*s. The Revert First Strand cDNA Synthesis kit (Thermo Fisher Scientific, USA) and an oligo-dT primer (Thermo Fisher Scientific, USA) were used to synthesize cDNA according to the manufacturer's instructions. For the RT-PCR, 300 ng of cDNA were used in a 10 µl reaction using the Phusion High-Fidelity DNA Polymerase (Thermo Fischer Scientific, USA) and 0.5 µM of TPS-specific primers (Supplementary Table S17). A cycling programme of 98°C for 30 sec, followed by 40 cycles of 98°C for 30 sec, 60°C for 20 sec, 72°C for 30 sec, with a final extension step at 72°C for 5 min was used. The amplicons were purified and send for sequencing to verify primer specificity. For the RT-qPCR, the cDNA was diluted 10 times (~300 ng $\mu l^{-1}$) and 2 µl were used as a template in a 10 µl reaction, using 5 µl of the Brilliant III Ultra-Fast SYBR® Green (Agilent Technologies, USA) and TPS-specific primers (Supplementary Table S17) in a concentration of 0.3-0.5 µM. Each RT-qPCR reaction was performed with three technical replications for the four biological replications. The RT-qPCR amplification was performed using the AriaMx Real-Time PCR System-G8830A (Agilent Technologies, USA) with a programme of 95°C for 5 min, followed by 40 cycles of 95°C for 30 sec, 60°C for 1 min, and 72°C for 1 min. A final dissociation step was performed to assess the quality of amplified products and the specificity of the primers. Expression of *TPS*s was normalised using *S. lycopersicum* β-tubulin (TUB, GenBank: DQ205342.1) expression levels. Calculation of gene expression ratio between inoculated and non-inoculated plants and statistical analyses were performed using the software tool REST© [91] (Supplementary Figure S4).
